# T2-Weighted MRI Radiomic Features Predict Prostate Cancer Presence and Eventual Biochemical Recurrence

**DOI:** 10.3390/cancers15184437

**Published:** 2023-09-06

**Authors:** Savannah R. Duenweg, Samuel A. Bobholz, Michael J. Barrett, Allison K. Lowman, Aleksandra Winiarz, Biprojit Nath, Margaret Stebbins, John Bukowy, Kenneth A. Iczkowski, Kenneth M. Jacobsohn, Stephanie Vincent-Sheldon, Peter S. LaViolette

**Affiliations:** 1 Department of Biophysics, Medical College of Wisconsin, 8701 Watertown Plank Rd., Milwaukee, WI 53226, USA; sduenweg@mcw.edu (S.R.D.); mstebbins@mcw.edu (M.S.); 2 Department of Radiology, Medical College of Wisconsin, 8701 Watertown Plank Rd., Milwaukee, WI 53226, USA; 3 Department of Electrical Engineering and Computer Science, Milwaukee School of Engineering, 1025 N Broadway, Milwaukee, WI 53202, USA; 4 Department of Pathology, Medical College of Wisconsin, 8701 Watertown Plank Rd., Milwaukee, WI 53226, USA; kaiczkowski@mcw.edu; 5 Department of Urology, Medical College of Wisconsin, 8701 Watertown Plank Rd., Milwaukee, WI 53226, USA; 6 Department of Biomedical Engineering, Medical College of Wisconsin, 8701 Watertown Plank Rd., Milwaukee, WI 53226, USA

**Keywords:** prostate cancer, mp-MRI, biochemical recurrence, Gleason pattern, radiomic features

## Abstract

**Simple Summary:**

Prostate cancer (PCa) is the leading non-cutaneous male cancer diagnosis in the United States. This study used radiomic features calculated from T2-weighted magnetic resonance imaging to predict biochemical recurrence (BCR) and PCa presence. A total of 279 patients (n = 46 BCR) undergoing imaging before surgery were analyzed for this study. Radiomic features were calculated for the whole prostate and within pathologist-annotated cancerous lesions. A tree regression model predicted BCR with AUC = 0.97, and a tree classification model classified PCa presence with 89.9% accuracy. This research demonstrates the feasibly of a radiomic features-based tool for screening PCa presence and metastatic risk in a clinical setting.

**Abstract:**

Prostate cancer (PCa) is the most diagnosed non-cutaneous cancer in men. Despite therapies such as radical prostatectomy, which is considered curative, distant metastases may form, resulting in biochemical recurrence (BCR). This study used radiomic features calculated from multi-parametric magnetic resonance imaging (MP-MRI) to evaluate their ability to predict BCR and PCa presence. Data from a total of 279 patients, of which 46 experienced BCR, undergoing MP-MRI prior to surgery were assessed for this study. After surgery, the prostate was sectioned using patient-specific 3D-printed slicing jigs modeled using the T2-weighted imaging (T2WI). Sectioned tissue was stained, digitized, and annotated by a GU-fellowship trained pathologist for cancer presence. Digitized slides and annotations were co-registered to the T2WI and radiomic features were calculated across the whole prostate and cancerous lesions. A tree regression model was fitted to assess the ability of radiomic features to predict BCR, and a tree classification model was fitted with the same radiomic features to classify regions of cancer. We found that 10 radiomic features predicted eventual BCR with an AUC of 0.97 and classified cancer at an accuracy of 89.9%. This study showcases the application of a radiomic feature-based tool to screen for the presence of prostate cancer and assess patient prognosis, as determined by biochemical recurrence.

## 1. Introduction

Prostate cancer (PCa) is the leading male cancer diagnosis in the United States and saw an incidence rate increase of 3% annually from 2014 through 2019. There is a lifetime probability of one in eight men developing invasive prostate cancer, and this constitutes an estimated 288,300 new male cancer diagnoses in 2023 [1]. Although PCa is expected to account for 27% of new male cancer diagnoses, not all cases have high metastatic potential or mortality risk. Despite this and improved screening and treatment, an estimated 20% to 30% of prostate cancer patients will experience biochemical recurrence (BCR) within five years of treatment [2,3,4].

Multiparametric magnetic resonance imaging (MP-MRI), including T2-weighted imaging (T2WI) and apparent diffusion coefficient (ADC) maps calculated from diffusion weighted imaging (DWI), has been used to assess PCa and has been a promising tool in diagnosing high-grade PCa [5,6,7]. The Prostate Imaging Reporting and Data System (PI-RADS) has standardized the acquisition, interpretation, and reporting of prostate MRI. PI-RADS has aided in the detection of cancerous lesions and has improved the consistency of clinical radiology reads [8,9,10,11]. While PI-RADS has improved cancer detection and is frequently used for biopsy guidance, it has not improved the stratification of patients at risk of BCR; therefore, there has been an increase in studies attempting to detect PCa with high metastatic potential [12,13,14].

PCa histology is graded using the Gleason grading scale, which assigns a score corresponding to the Gleason grades of the two most prevalent cell patterns. These patterns have more recently been used to assign patients into one of five Grade Groups (GG) to predict prognosis [15]. Low risk cancers, detected through biopsies, may be managed through active surveillance using prostate specific antigen (PSA) blood tests. Clinically significant cancer (GG ≥ 2, tumor volume ≥ 0.5 mL, or clinical stage ≥ T3) is more often treated with radical prostatectomy, or removal of the prostate, and/or radiation. Prostatectomy is considered curative if tumors are organ-confined; however, distant metastases may form and result in biochemical recurrence. Currently, PSA is the only validated biomarker for BCR [2], which is often defined as a rise in PSA from 0 ng/mL after surgery to more than 0.1 or 0.2 ng/mL [3,4]. Improved strategies for predicting the biochemical recurrence of PCa have been increasingly assessed in pathological studies; however, few studies using MRI-based features have been used to noninvasively predict BCR. Previous studies have modeled MRI features such as the PI-RADSv2.1 score, MRI lesion volume/percentage, extraprostatic extension, and seminal vesicle invasion with respect to BCR; however, these features are all well-known to be related to BCR-risk [16,17].

Radiomics is a multi-step process where quantitative features or textures from MR images are extracted using data-characterization algorithms to decode underlying tissue characteristics [7,8]. Radiomic features contain first-, second-, and higher-order statistics that are combined with other patient data to develop models that may detect and characterize a clinically relevant outcome [9,10,11,12]. They are thought to capture distinct phenotypic differences in tumors and quantitatively assess intra- and intertumoral heterogeneity [18,19]. Recent radiomic feature analyses in prostate cancer research have assessed their ability to differentiate low from higher-grade prostate cancer [20,21,22,23], predict Gleason Grades [24,25,26], and identify tumor presence [18,19]. Radiomic feature calculations are highly sensitive to variations in image acquisition and often sacrifice the interpretability of these mathematical representations of image characteristics; however, if properly used as inputs to machine and deep learning models, they can non-invasively identify relationships and potential biomarkers of a myriad of diseases [27,28,29,30,31].

While PI-RADS and Gleason Grade Groups are the two current gold-standard metrics in assessing prostate cancer severity and risk, both scores are assigned qualitatively and are subjected to interrater variability, which can lead to the overtreatment of low-risk cancers [9,32,33,34,35,36]. With the promising applications of radiomic features in assessing prostate cancer, this study sought to determine not only whether quantitative radiomic features of prostate cancer MRI differ between regions of cancer and non-cancer on T2-weighted imaging, but if these features could predict cancer severity and metastatic risk. Specifically, we tested the hypothesis that a radiomic feature-based model could be utilized as a non-invasive screening tool for prostate cancer presence and risk, detecting tumoral regions and biochemical recurrence risk. Additionally, we sought to determine whether these features could be applied to a simulated low-resolution “quick” MRI that could be clinically practiced for routine patient care.

## 2. Materials and Methods

### 2.1. Patient Population and Data Acquisition

Data from 279 prospectively recruited patients with biopsy-confirmed prostate cancer who underwent radical prostatectomy between 2014 and 2023 were analyzed for this institutional review board (IRB) approved study. Patients underwent multi-parametric magnetic resonance imaging (MP-MRI) prior to surgery on a 3T MRI scanner (General Electric, Waukesha, WI, USA or Siemens Healthineers, Erlangen, Germany) with or without an endorectal coil. Each protocol included T2-weighted imaging (T2WI), dynamic contrast enhanced imaging (DCE), and diffusion-weighted imaging (DWI), though this study focused on T2WI, due to the lack of acquisition parameter changes between PI-RADS v2.0 and v2.1. The following are example T2W acquisition parameters: repetition time = 4500 ms; 512 × 512 voxel acquisition matrix; 120 mm field of view (FOV); 3 mm slice thickness; 0.234 × 0.234 mm voxel dimensions; and 30 slices/acquisition (range 16–44). All T2WI were intensity normalized using the z-score of intensity within the mask of the prostate.

Following prostatectomy, patients were monitored with prostate-specific antigen (PSA) testing according to standard of care practices (mean 2.1 years, range 0.1–7.3 years). Patients with a measured PSA score of ≥0.2 ng/mL at any timepoint following surgery were considered biochemically recurrent [37,38]. Inclusion criteria for this study included T2-weighted imaging and at least one post-surgery PSA test. A subset of 109 patients with digitized and pathologist-provided Gleason pattern annotated histology was used to assess the radiomic features of cancerous and non-cancerous regions on the MRI. Patient demographic information and clinical features for the whole study cohort are summarized in Table 1, and the subset cohort in Supplemental Table S1.

### 2.2. Histological Analysis

**Figure 1:**
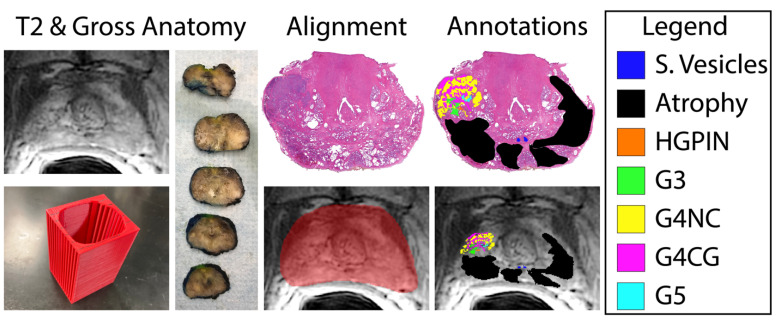
Tissue processing and MRI co-registration process. Slicing jigs (**bottom**, **left**) are 3D modeled to section tissue in line with the slice thickness and orientation of each patient’s T2-weighted image (**left**). Slides are digitized and annotated by Gleason pattern (legend, (**right**)) and aligned to the corresponding slice on the T2WI (**bottom**, **middle**).

Prostatectomy was performed following imaging using the da Vinci robotic system (Intuitive Surgical, Sunnyvale, CA, USA) [39,40]. Prior to 2020, prostatectomy occurred approximately 4 weeks after imaging; however, since the COVID-19 pandemic, the time between imaging and surgery has increased to approximately 19 weeks. Prostate samples were formalin-fixed overnight and sectioned using a custom, 3D printed slicing jig modeled after the orientation and slice thickness of the T2WI [41]. Briefly, prostate masks were manually drawn using AFNI (Analysis of Functional NeuroImages, http://afni.nimh.nih.gov/, accessed on 5 April 2019) [42], 3D modeled using 3dSlicer (https://slicer.org, accessed on 20 December 2017), and imported into Blender 2.75 (https://www.blender.org/, accessed on 22 March 2018) to create the final slicing jig [35,43,44,45], and finally 3D printed using a fifth-generation Makerbot (Makerbot Industries, Brooklyn, NY, USA) (Figure 1, left).

Whole-mount tissue sections were paraffin-embedded, axially sectioned, and hematoxylin and eosin (H&E)-stained in our histology core lab. Stained slides were digitally scanned at 40× magnification using either Nikon (Nikon Metrology, Brighton, MI, USA), Olympus (Olympus Corporation, Tokyo, Japan), or Huron (Huron Digital Pathology, Ontario, Canada) sliding stage microscopes at resolutions of 0.8, 0.34, or 0.2 microns per pixel, respectively. Multiple slide scanners were used due to upgrading equipment throughout the evolution of our lab. Slides were down sampled by a factor of 8 for the Nikon or Olympus scanners or 10 for the Huron slide scanner, to account for the large file size of the raw, scanned slides and the increased resolution of the Huron scanned images. Scanned slides were annotated by a board-certified genitourinary pathologist to identify regions of unique Gleason patterns, including Gleason 3 (G3), G4 non-cribriform glands (G4NC), G4 cribriform (to papillary) glands (G4CG), and Gleason 5 (G5), as well as non-cancerous regions including seminal vesicles, atrophy, and high-grade prostatic intraepithelial neoplasia (HGPIN). Gleason 4 patterns were separately annotated due to their prognostic differences [34,46,47,48,49]; however, all cancerous annotations were combined into one class for these analyses. An example of these annotations can be found in Figure 1.

### 2.3. Histology and MRI Co-Registration

Digitized whole-slide images, as well as their respective annotation masks, were co-registered to the T2-weighted image using previously published in-house software and techniques [31,35,41,43,44,45,50,51] (*n* = 3 slides/patient). Briefly, slides were flipped and rotated to match their respective MRI slice, and a control-point co-registration was applied using manually fixed points corresponding to their respective position between both modalities. Specifically, points were arranged along the boundaries of and on distinct landmarks within the prostate (i.e., the urethra, seminal vesicles, etc.). Approximately 20–30 control points were placed on each slide, which was then down-sampled to MRI resolution. A nonlinear, spatial transform was calculated from control points using Matlab 2021b (The MathWorks, Natick, MA, USA) “fitgeotrans” function and applied using the “imwarp” function. This software allows for the histology and resulting pathologist-assessment to be compared one-to-one with clinical in vivo images by using a local weighted-means transform to account for non-uniform distortions. An additional nearest-neighbor interpolation was applied on annotations to retain integer values (Figure 1, right).

### 2.4. Radiomic Feature Extraction and Statistical Analyses

Radiomic features were extracted using Pyradiomics v3.1.0 [52] in Visual Studio Code v1.79, including 14 shape features, 18 first-order features, and 75 higher-order features. These features were extracted first across the whole prostate within the prostate mask, and then again for cancerous and non-cancerous regions of interest, delineated through our pathologist’s annotations and normalized using the training data’s Z-score for each feature. To prevent model overfitting, a *t*-test was performed to determine features that were significantly different between (1) patients who did and did not biochemically recur and (2) within regions of cancer and non-cancer, and these features were used for model input parameters.

Both patient cohorts were divided using a 2/3rd–1/3rd train/test split, balancing for Gleason Grade and biochemical recurrence. Two fine tree models, which allowed for greater splits and thus fine distinctions between classes, were trained using Matlab 2021b with a 5-fold cross-validation to (1) predict patients who will experience eventual biochemical recurrence and (2) classify regions of cancer from non-cancer. Models were evaluated on the withheld validation set and test set using the area under the receiver operating characteristic (ROC AUC) curve for the BCR regression model and accuracy of the cancer/non-cancer classification model.

## 3. Results

### 3.1. Biochemical Recurrence Regression Model

**Figure 2:**
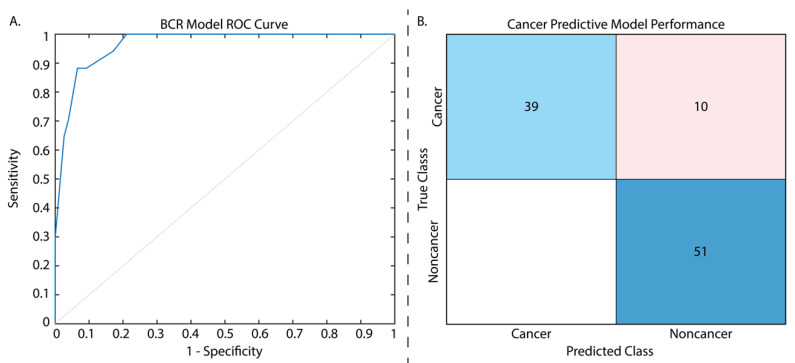
Results from the two tested models. The ROC curve presented for the biochemical recurrence regression model (**A**) has an AUC = 0.97. The cancer classification model confusion matrix (**B**) is displayed with normalized classification rates (accuracy = 89.9%). Confusion matrix legend: blue = true positive, red = false positive.

From our *t*-test analysis, of the initial 107 calculated radiomic features, only 7 first-order features were found to be significantly different between patients who did and did not experience eventual BCR (*p* < 0.01), including the following: 90th percentile, Interquartile Range, Mean, Mean Absolute Deviation, Robust Mean Absolute Deviation, Root Mean Squared, and Variance. An additional two first-order features, Maximum and Range, and four higher-order, gray level size zone (GLSZM) features (i.e., Size Zone Non-Uniformity Normalized, Small Area Emphasis, Small Area High Gray Level Emphasis, and Zone Entropy) saw trending significance (all *p* < 0.1). Of the latter features, only Zone Entropy aided in model performance, and thus the remaining features were excluded from the final model. The final features used in this model are described in Table 2, with the full assessment in Supplemental Table S2, and parameters are detailed in Supplemental Table S3. This regression model was trained on data from 186 patients, and when evaluated on the withheld test set of 93 patients had a testing AUC of 0.97. Defining a cutoff that maximized the sum of sensitivity and specificity in the test set gave us a peak sensitivity of 0.88, a specificity of 0.93, and an accuracy of 75% (Figure 2A; Table 3).

### 3.2. Cancer/Non-Cancer Classification Model

From our *t*-test analysis, only 13 features of the 107 were not found to have significantly different cancerous and non-cancerous regions, and thus we chose to use the same 10 features used for our regression analysis in this model to determine whether these features alone could determine BCR risk and cancer presence and prevent model overfitting. T-test results for radiomic features between cancer and non-cancer can be found in Supplemental Table S4, and classification model parameters in Table 3. The cancer classification model was assessed on the validation set during model training and again on the withheld test set (*n* = 74 patients), and had an accuracy of 90.7% and 89.9%, respectively, sensitivity of 0.80, specificity of 1, and AUC of 0.95 (Figure 2B; Table 3).

### 3.3. Low-Resolution Image Assessment

**Figure 3:**
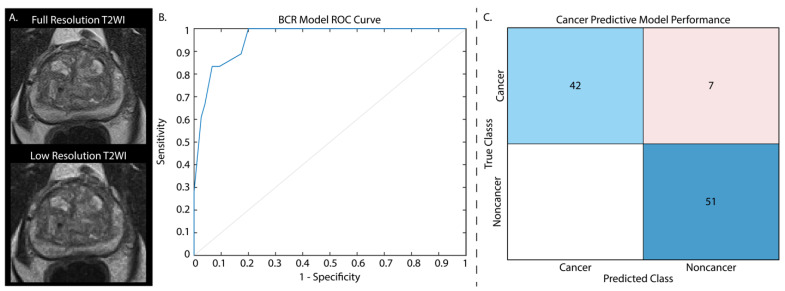
T2-weighted images from the withheld test set were down-sampled by a factor of two and noise was added to each image (**A**). Cancer classification performance is displayed as normalized values. Applying our BCR model to these noisy images had an AUC = 0.96 (**B**). The cancer classification model confusion matrix (**C**) is displayed with normalized classification rates (accuracy = 92.8%).

As these models showed high performance at assessing cancer presence and BCR risk, we further assessed their capabilities on artificially noisy data as a proof of concept to determine whether this model could be applied to low-quality images that may be rapidly acquired as a prostate cancer screening tool. Briefly, simulated Gaussian noise was added to the testing data with a mean centered on 50 and a variance of 100, and furthermore down-sampled by a factor of 2 (Figure 3A). Whole prostate, cancer, and non-cancer annotation masks were likewise down-sampled to retain the same space on the low quality images, and radiomic features were re-extracted. These features were normalized using the full resolution training data Z-score for each feature, and the same independent test sets that were used in the previous applications were again assessed. Applying the regression model to predict BCR yielded an AUC = 0.96 (Figure 3B) and the classification model to the low-resolution test data yielded a test accuracy of 92.8%, highlighting the ability of these radiomic features in detecting cancer on poor quality images (Figure 3C) and assessing BCR risk.

## 4. Discussion

Due to the significant rate of prostate cancer recurrence, there has been increasing interest in accurately and promptly predicting biochemical recurrence. This interest stems from the need to identify individuals who are at a high risk of experiencing adverse outcomes. Although the likelihood of BCR is dependent on the extent and aggressiveness of the cancer, roughly 20 percent to 30 percent of men will relapse within five years post treatment [53,54]. These trends were observed in our cohort, where 16% of patients experienced BCR despite an average follow-up time of 2 years. Currently, the current gold standard prognostic indicator of BCR is defined by Gleason Grade Groups and measured with increased PSA, which is monitored post radical prostatectomy [55,56,57]. Non-invasive metrics to predict prostate cancer BCR risk are not widely researched and may hinder patient treatment and follow-up following surgery.

This study showed that the use of quantitative radiomic features calculated from T2-weighted MR imaging has the potential to offer supplementary information beyond the conventional pathological evaluation. Specifically, we trained a tree regression model using nine first-order and one higher-order radiomic features calculated and averaged across the whole prostate to predict BCR, which had AUC = 0.97 and an accuracy of 75% when defining a cutoff to maximize sensitivity and specificity in the test set. These results are especially promising given the imbalance between recurrent and non-recurrent groups. Ideally, these two groups would be balanced in the training data; however, our imbalanced cohorts followed similar trends as the real-world recurrence rate and may better generalize to the natural trend. Similar studies using radiomic features to predict biochemical recurrence likewise did not balance these groups and saw comparable or lower classification accuracies and/or AUC to the present study [58,59,60].

Additionally, we found that these same features when used as the input to a tree classification model could accurately identify regions of cancer from non-cancer on T2WI alone. The quantitative characteristics extracted from prostate cancer MR imaging have the potential to assist clinicians in predicting a patient’s likelihood of biochemical recurrence, surpassing the insights gained from a qualitative analysis of gland morphology, as used in the Gleason grading scale. The models used in this study primarily used MRI intensity metrics, which may indicate that intensity can not only stratify cancer aggressiveness, as described by the PI-RADS grading system, but may be an additional characteristic to stratify patient prognosis. This was especially noted when using imaging that was down-sampled and with additional simulated noise. Using our models on radiomic features calculated across these low-resolution images provided high accuracy in both determining cancer presence and assigning metastatic potential. These results on low-resolution images highlighted the feasibility of using these models as a non-invasive prostate cancer screening tool, similar in nature to a woman’s annual mammogram. A screening tool such as this may detect prostate cancer before it has progressed to a stage requiring surgical intervention.

Previous studies have explored the use of radiomic feature-based models in predicting prostate cancer [30,61,62]. These models leverage the quantitative analysis of radiographic images to extract a wide range of features, such as shape, texture, and intensity, which can provide valuable insights into tumor characteristics. By incorporating these radiomic features into predictive models, researchers have aimed to enhance the accuracy and precision of prostate cancer diagnosis, risk stratification, and prognosis prediction. Likewise, in our current study, we have shown the added benefit of assessing a limited number of MRI-based features to non-invasively predict prostate cancer presence and severity. Previous studies showed 64 percent to 82 percent accuracy at annotated Gleason patterns using radiomic features calculated from T2WI and ADC maps [24,63]. While our current study did not assess the ability of our features to predict exact Gleason patterns, and was limited to using only T2WI, these studies show the potential of radiomic features in prostate cancer screening. Additionally, previous studies utilizing radiomic features to predict BCR showed similar success [64,65]. Algohary et al. found that the inclusion of radiomic features from regions radiologically delineated for low-, intermediate-, and high-risk regions on T2WI and ADC and pathologically defined on whole slide images improved risk stratification by 3–6%; however, this study did not have precise histology–MRI alignment [64]. Additionally, their model, while achieving an AUC of 0.87, only had an overall accuracy of 53%. Another recent study by Lee et al. found that radiomic features from manually delineated index tumors on T2WI, ADC, and contrast-enhanced imaging (DCE) in combination with clinical variables saw increased performance using a Cox model compared to clinical variables or conventional radiomics alone [66]. Our current models were not only successful in predicting metastatic potential, they also did not require a manually delineated tumoral region, as in these previous studies. These models have shown promising results, demonstrating their potential as non-invasive tools for assessing cancer aggressiveness, treatment response, and patient outcomes.

In this study, we tested the hypothesis that radiomic features of prostate cancer can predict eventual biochemical recurrence and tumor presence. Of the 107 total radiomic features calculated, we found 7 first-order features that significantly differed between patients who did and did not experience recurrence following radical prostatectomy, and an additional 3 features with marginal significance that improved our model’s accuracy. While most radiomic features were significantly different between regions of cancer and non-cancer, we found that the utilization of the aforementioned features could reliably identify cancerous lesions. These findings suggest that the variation in MRI-based features may be correlated with the aggressiveness and prognosis of prostate cancer.

### Limitations

Although the findings of this study are propitious, it is essential to acknowledge several limitations. In comparison to previous studies on prostate cancer, this study utilizes a relatively small patient cohort with a short average follow-up time, which may bias it towards identifying rapidly recurring patients. As a result, employing a larger cohort of patients followed for longer may improve the generalizability to external data, as well as to late-recurring patients. Furthermore, though our patient cohort fell slightly below the average recurrence rate of approximately 20%, the imbalance between recurrent and non-recurrent groups in our study may have led our models to underpredict BCR and thus should be additionally validated with a secondary patient cohort. Additionally, though this was a single-center study, only clinical imaging was examined; therefore, there was a lack of standardization in acquisition parameters. This may confound future studies using imaging from additional MR vendors. Future studies should compare radiomic features calculated from other clinical MR vendors and imaging sequences to more precisely model BCR risk. Furthermore, only mean whole-prostate and lesion-wise radiomic features were investigated in our models; thus, future studies should examine voxel-wise or larger tile-based radiomic features. Additionally, images both using and not using an endorectal coil may have caused a bias to our analyses as the endorectal coil is known to not only alter the prostatic surface, but also impact the MRI signal. While it has been debated whether the MR intensity signal is significantly impacted by endorectal coil usage [67,68,69,70], it still may have impacted the results of our study.

The use of multiple slide scanners of varying resolutions, and thus different down-sampling factors, may be an additional confounding factor in our analyses. The increased down-sampling factor for the slides scanned on the Huron slide scanner was used to reduce the high-resolution images to approximately match the resolution of the other two slide scanners; however, these changes in digital images may have impacted our pathologist’s ability to distinguish unique Gleason patterns. Finally, pathologist-provided cancer annotations were used as a ground truth for the cancerous region model, which may not best inform a radiologist’s delineation of cancer on MR imaging. Future studies should additionally assess radiomic features based on radiologist-annotated tumoral regions.

## 5. Conclusions

We demonstrated that in a cohort of 279 patients with biopsy-confirmed prostate cancer, radiomic features were able to predict eventual biochemical recurrence. Additionally, in a subset of 109 patients, these same radiomic features were able to classify regions of cancer from non-cancer at the whole-lesion level. These features may be quickly extracted from patients’ T2-weighted MR images to determine whether cancer is present and aid in patient monitoring after treatment. Future studies should assess these features in larger patient cohorts to further develop predictive models and determine if higher-grade cancers can be further circumscribed in prostate MRI.

## Figures and Tables

**Table 1:** Summary of patient information across the prostate cancer cohort at the time of surgery.

Clinical Feature	Training (*n* = 186)	Testing (*n* = 93)	Total (*n* = 279)
Age at RP, years (mean ± SD)	62 ± 6	62 ± 7	62 ± 6
Race (*n*, %)			
African American	11 (6)	5 (5)	16 (6)
White/Caucasian	91 (49)	43 (46)	134 (48)
Asian	12 (6)	5 (5)	17 (6)
Other	3 (2)	0 (0)	3 (1)
Missing	80 (43)	45 (48)	125 (45)
Preoperative PSA, ng/mL (*n*, %)			
<6	77 (41)	50 (54)	127 (46)
≥6–10	71 (38)	23 (25)	94 (34)
≥10–20	31 (17)	16 (17)	47 (17)
≥20–30	5 (3)	2 (2)	7 (3)
≥30	2 (1)	2 (2)	4 (1)
Grade group at RP (*n*, %)			
6	18 (10)	10 (11)	28 (10)
3 + 4	117 (63)	52 (56)	169 (61)
4 + 3	26 (14)	15 (16)	41 (15)
8	5 (3)	4 (4)	9 (3)
≥9	20 (11)	12 (13)	32 (11)
Clinical Stage (*n*, %)			
T1	145 (78)	60 (65)	205 (73)
T2	24 (13)	22 (24)	46 (16)
Missing	11 (6)	17 (18)	28 (11)
Surgical Stage (*n*, %)			
2a,b	102 (55)	48 (52)	150 (54)
2c	29 (16)	12 (13)	41 (15)
3a,b	52 (28)	31 (33)	83 (30)
Missing	3 (1)	2 (2)	5(1)
Cribriform Pattern (*n*, %) (*n* = 257)			
Presence	75 (40)	40 (43)	115 (41)
Absence	98 (53)	44 (47)	142 (51)
Missing	13 (7)	9 (10)	22 (8)
Biochemical Recurrence (*n*, %)	28 (15)	18 (19)	46 (16)
Time to BCR, years (mean, range) (*n* = 45)	1.68 (0.1–5.0)		
Follow-up time post RP, years (mean, range)	2.1 (0.1–7.3)		

Abbreviations: RP = radical prostatectomy, PSA = prostate specific antigen, BCR = biochemical recurrence.

**Table 2:** Radiomic features used for model training. All *p*-values provided from a two-sample *t*-test between patients who did and did not experience BCR.

Class	Radiomic Features	t-Value	*p*-Value
First Order Features	90th Percentile	2.38	0.02 *
	Interquartile Range	2.07	0.04 *
	Maximum	−1.87	0.06 ^+^
	Mean	2.31	0.02 *
	Mean Absolute Deviation	2.16	0.03 *
	Range	−1.92	0.06^+^
	Robust Mean Absolute Deviation	2.25	0.03 *
	Root Mean Squared	2.05	0.04 *
	Variance	2.09	0.04 *
GLSZM	Zone Entropy	−1.88	0.06 ^+^

* *p* < 0.05. ^+^ 0.05 < *p* < 0.1.

**Table 3:** Performance metrics for both the BCR and cancer/non-cancer radiomic feature models.

Model	Accuracy	Sensitivity	Specificity
BCR (Full-Res)	AUC = 0.97	0.88	0.93
Cancer/Non-cancer (Full-Res)	ACC = 89.9%	0.80	1

## Data Availability

The data presented in this study are available on request from the corresponding author. The data are not publicly available due to privacy concerns.

## Supplementary Materials

The following supporting information can be downloaded at: https://www.mdpi.com/article/10.3390/cancers15184437/s1, Table S1: Demographics information for the subset of patients analyzed for cancer and non-cancer classification; Table S2: Extracted radiomic features from Pyradiomics; Table S3: Model parameters for both the BCR and cancer/non-cancer tree models; Table S4: Extracted radiomic features from Pyradiomics.
