# Supplementary material for: T2-Weighted MRI Radiomic Features Predict Prostate Cancer Presence and Eventual Biochemical Recurrence
Source: Cancers (Basel). 2023 Sep 6;15(18):4437. doi: 10.3390/cancers15184437 (PMC10526331; doi:10.3390/cancers15184437)
Supplement: Supplementary file 1 [file cancers-15-04437-s001.zip › cancers-2509993-supplementary.pdf]

Supplementary Table S1: Demographics information for the subset of patients analyzed for cancer and noncancer classification.

	Training (n = 74)	Testing (n = 35)	Total (n = 109)
Age at RP, years (mean ± SD)	60 ± 6	60 ± 7	60 ± 6
Race (n, %) (n = 101)			
African American	8 (4)	3 (3)	11 (10)
White/Caucasian	52 (28)	24 (26)	76 (70)
Asian	9 (5)	3 (3)	12 (11)
Other	2 (1)	0 (0)	2 (2)
Preoperative PSA, ng/mL (n, %)			
< 6	36 (19)	21 (23)	57 (52)
≥ 6 – 10	25 (13)	8 (9)	33 (30)
≥ 10 – 20	12 (6)	5 (5)	17 (16)
≥ 20 – 30	1 (1)	1 (1)	2 (2)
Grade group at RP (n, %)			
6	14 (8)	8 (9)	22 (20)
3+4	40 (22)	15 (16)	55 (50)
4+3	10 (5)	5 (5)	15 (14)
8	3 (2)	1 (1)	4 (4)
≥ 9	7 (4)	6 (6)	13 (12)
Clinical Stage (n, %)			
T1	68 (37)	23 (25)	91 (83)
T2	6 (3)	12 (13)	18 (17)
Surgical Stage (n, %) (n = 274)			
2a,b	23 (12)	9 (10)	32 (29)
2c	24 (13)	11 (12)	35 (32)
3a,b	27 (15)	15 (16)	42 (39)
Cribriform Presence (n, %) (n = 257)	18 (10)	14 (15)	32 (29)

Supplemental Table S2. Extracted radiomic features from Pyradiomics. A two-sample t-test was performed to assess significance between features in the BCR regression analysis. *Abbreviations: GLCM = Gray Level Co-occurrence Matrix, GLRLM = Gray Level Run Length Matrix, GLSZM = Gray Level Size Zone Matrix, NGTDM = Neighbouring Gray Tone Difference Matrix, GLDM = Gray Level Dependence Matrix.*

Class	Radiomic Feature	p-value
First Order	10Percentile	0.37
	90Percentile	0.02[*]
	Energy	0.37
	Entropy	0.87
	InterquartileRange	0.04[*]
	Kurtosis	0.22
	Maximum	0.06[+]
	Mean	0.02[*]
	MeanAbsoluteDeviation	0.03[*]
	Median	0.98
	Minimum	0.65
	Range	0.06[+]
	RobustMeanAbsoluteDeviation	0.03[*]
	RootMeanSquared	0.04[*]
	Skewness	0.21
	TotalEnergy	0.75
	Uniformity	0.87
	Variance	0.04[*]
Shape	Elongation	0.17
	Flatness	0.17
	LeastAxisLength	0.28
	MajorAxisLength	0.80
	Maximum2DDiameterColumn	0.68
	Maximum2DDiameterRow	0.43
	Maximum2DDiameterSlice	0.72
	Maximum3DDiameter	0.57
	MeshVolume	0.75
	MinorAxisLength	0.35
	Sphericity	0.14
	SurfaceArea	0.95
	SurfaceVolumeRatio	0.52
	VoxelVolume	0.75
GLCM	Autocorrelation	0.98
	ClusterProminence	0.53
	ClusterShade	0.76
	ClusterTendency	0.56
	Contrast	0.57

	Correlation	0.55
	DifferenceAverage	0.57
	DifferenceEntropy	0.45
	DifferenceVariance	0.48
	Id	0.57
	Idm	0.57
	Idmn	0.57
	Idn	0.57
	Imc1	0.43
	Imc2	0.59
	InverseVariance	0.57
	JointAverage	0.93
	JointEnergy	0.55
	JointEntropy	0.49
	MCC	0.55
	MaximumProbability	0.79
	SumAverage	0.93
	SumEntropy	0.45
	SumSquares	0.95
GLDM	DependenceEntropy	0.31
	DependenceNonUniformity	0.26
	DependenceNonUniformityNormalized	0.34
	DependenceVariance	0.59
	GrayLevelNonUniformity	0.38
	GrayLevelVariance	0.87
	HighGrayLevelEmphasis	0.76
	LargeDependenceEmphasis	0.76
	LargeDependenceHighGrayLevelEmphasis	0.73
	LargeDependenceLowGrayLevelEmphasis	0.83
	LowGrayLevelEmphasis	0.76
	SmallDependenceEmphasis	0.34
	SmallDependenceHighGrayLevelEmphasis	0.27
	SmallDependenceLowGrayLevelEmphasis	0.45
GLRLM	GrayLevelNonUniformity	0.46
	GrayLevelNonUniformityNormalized	0.36
	GrayLevelVariance	0.36
	HighGrayLevelRunEmphasis	0.37
	LongRunEmphasis	0.47
	LongRunHighGrayLevelEmphasis	0.50
	LongRunLowGrayLevelEmphasis	0.45
	LowGrayLevelRunEmphasis	0.37
	RunEntropy	0.42
	RunLengthNonUniformity	0.49

	RunLengthNonUniformityNormalized	0.85
	RunPercentage	0.76
	RunVariance	0.49
	ShortRunEmphasis	0.69
	ShortRunHighGrayLevelEmphasis	0.53
	ShortRunLowGrayLevelEmphasis	0.98
GLSZM	GrayLevelNonUniformity	0.37
	GrayLevelNonUniformityNormalized	0.23
	GrayLevelVariance	0.23
	HighGrayLevelZoneEmphasis	0.69
	LargeAreaEmphasis	0.77
	LargeAreaHighGrayLevelEmphasis	0.72
	LargeAreaLowGrayLevelEmphasis	0.80
	LowGrayLevelZoneEmphasis	0.69
	SizeZoneNonUniformity	0.91
	SizeZoneNonUniformityNormalized	0.07^{+}
	SmallAreaEmphasis	0.06^{+}
	SmallAreaHighGrayLevelEmphasis	0.07^{+}
	SmallAreaLowGrayLevelEmphasis	0.15
	ZoneEntropy	0.06^{+}
	ZonePercentage	0.49
	ZoneVariance	0.77
NGTDM	Busyness	0.90
	Coarseness	0.64
	Complexity	0.42
	Contrast	0.44
	Strength	0.64

* $p < 0.05$
$^{+}$ $0.05 < p < 0.1$

Supplemental Table S3. Model parameters for both the BCR and Cancer/Noncancer tree models.
Abbreviations: MSE = Mean Squared Error, GDI = Dini's diversity index.

**Property**	**BCR - Value**	**Cancer - Value**
Type	Regression	Classification
Split Criterion	MSE	GDI
Min. Number of Branch Node Observations	10	10
Min. Number of Leaf Node Observations	4	1
Max. Number of Decision Splits	278	100
Prune Criterion	MSE	Error
Predictor Selection	All Splits	All Splits

Supplemental Table S4. Extracted radiomic features from Pyradiomics. A two-sample t-test was performed to assess significance between features in the cancer/noncancer classification analysis. *Abbreviations: GLCM = Gray Level Co-occurrence Matrix, GLRLM = Gray Level Run Length Matrix, GLSZM = Gray Level Size Zone Matrix, NGTDM = Neighbouring Gray Tone Difference Matrix, GLDM = Gray Level Dependence Matrix.*

Class	Radiomic Feature	p-value
First Order	10Percentile	4.73E-19***
	90Percentile	1.82E-06***
	Energy	6.00E-70***
	Entropy	5.31E-14***
	InterquartileRange	7.94E-15***
	Kurtosis	0.35
	Maximum	1.60E-38***
	Mean	0.66
	MeanAbsoluteDeviation	1.77E-21***
	Median	0.34
	Minimum	2.01E-34***
	Range	1.90E-46***
	RobustMeanAbsoluteDeviation	4.51E-16***
	RootMeanSquared	1.60E-08***
	Skewness	0.25
	TotalEnergy	3.19E-63***
	Uniformity	3.34E-16***
	Variance	1.71E-24***
Shape	Elongation	3.37E-20***
	Flatness	0.02*
	LeastAxisLength	2.23E-28***
	MajorAxisLength	1.22E-16***
	Maximum2DDiameterColumn	1.04E-41***
	Maximum2DDiameterRow	9.85E-48***
	Maximum2DDiameterSlice	1.03E-35***
	Maximum3DDiameter	2.62E-34***
	MeshVolume	9.45E-68***
	MinorAxisLength	6.63E-55***
	Sphericity	7.65E-14***
	SurfaceArea	3.47E-72***
	SurfaceVolumeRatio	4.82E-36***
	VoxelVolume	1.04E-67***
GLCM	Autocorrelation	0.14
	ClusterProminence	1.78E-04***
	ClusterShade	0.96
	ClusterTendency	5.20E-20***
	Contrast	1.26E-06***

	Correlation	0.55
	DifferenceAverage	1.26E-06***
	DifferenceEntropy	2.89E-14***
	DifferenceVariance	1.78E-14***
	Id	1.26E-06***
	Idm	1.26E-06***
	Idmn	1.26E-06***
	Idn	1.26E-06***
	Imc1	0.17
	Imc2	4.24E-04***
	InverseVariance	1.26E-06***
	JointAverage	0.06+
	JointEnergy	8.54E-22***
	JointEntropy	2.30E-21***
	MCC	4.83E-26***
	MaximumProbability	0.86
	SumAverage	0.06+
	SumEntropy	4.03E-23***
	SumSquares	1.46E-21***
GLDM	DependenceEntropy	4.77E-24***
	DependenceNonUniformity	4.15E-56***
	DependenceNonUniformityNormalized	7.76E-07***
	DependenceVariance	7.44E-52***
	GrayLevelNonUniformity	3.53E-74***
	GrayLevelVariance	3.34E-16***
	HighGrayLevelEmphasis	0.19
	LargeDependenceEmphasis	2.25E-72***
	LargeDependenceHighGrayLevelEmphasis	4.15E-61***
	LargeDependenceLowGrayLevelEmphasis	4.98E-48***
	LowGrayLevelEmphasis	0.19
	SmallDependenceEmphasis	4.75E-35***
	SmallDependenceHighGrayLevelEmphasis	1.95E-35***
	SmallDependenceLowGrayLevelEmphasis	3.54E-24***
GLRLM	GrayLevelNonUniformity	2.26E-75***
	GrayLevelNonUniformityNormalized	2.08E-11***
	GrayLevelVariance	2.08E-11***
	HighGrayLevelRunEmphasis	0.29
	LongRunEmphasis	1.26E-26***
	LongRunHighGrayLevelEmphasis	2.26E-26***
	LongRunLowGrayLevelEmphasis	7.25E-21***
	LowGrayLevelRunEmphasis	0.29
	RunEntropy	2.30E-12***
	RunLengthNonUniformity	2.24E-58***

	RunLengthNonUniformityNormalized	2.67E-08***
	RunPercentage	1.54E-04***
	RunVariance	4.50E-32***
	ShortRunEmphasis	5.76E-04***
	ShortRunHighGrayLevelEmphasis	0.004**
	ShortRunLowGrayLevelEmphasis	0.003**
GLSZM	GrayLevelNonUniformity	7.08E-31***
	GrayLevelNonUniformityNormalized	0.02*
	GrayLevelVariance	0.02*
	HighGrayLevelZoneEmphasis	0.002**
	LargeAreaEmphasis	1.33E-50***
	LargeAreaHighGrayLevelEmphasis	1.35E-47***
	LargeAreaLowGrayLevelEmphasis	1.39E-46***
	LowGrayLevelZoneEmphasis	0.002**
	SizeZoneNonUniformity	9.24E-26***
	SizeZoneNonUniformityNormalized	1.21E-04***
	SmallAreaEmphasis	1.67E-08***
	SmallAreaHighGrayLevelEmphasis	3.79E-07***
	SmallAreaLowGrayLevelEmphasis	4.78E-05***
	ZoneEntropy	2.51E-17***
	ZonePercentage	9.78E-13***
	ZoneVariance	7.79E-53***
NGTDM	Busyness	3.29E-04***
	Coarseness	0.02*
	Complexity	1.97E-29***
	Contrast	3.19E-34***
	Strength	2.97E-07***

* $p < 0.05$
** $0.001 < p < 0.01$
*** $p < 0.001$
$^{+}$ $0.05 < p < 0.1$
